# Anticancer Activity of Paclitaxel-Loaded Mesoporous Silica Nanoparticles in B16F10 Melanoma-Bearing Mice

**DOI:** 10.3390/pharmaceutics17081042

**Published:** 2025-08-11

**Authors:** Jihoon Lee, Jung Mo Kim, Yeon-Ju Baek, Hyojeung Kang, Min-Koo Choi, Im-Sook Song

**Affiliations:** 1 BK21 FOUR Community-Based Intelligent Novel Drug Discovery Education Unit, Vessel-Organ Interaction Research Center (VOICE), College of Pharmacy and Research Institute of Pharmaceutical Sciences, Kyungpook National University, Daegu 41566, Republic of Korea; legadema0905@knu.ac.kr (J.L.); kjm3100@naver.com (J.M.K.); hkang72@knu.ac.kr (H.K.); 2 College of Pharmacy, Dankook University, Cheonan 31116, Republic of Korea; sjh72489@naver.com; 3 School of Pharmacy and Pharmaceutical Sciences, University of California, Irvine, CA 92697, USA

**Keywords:** paclitaxel (PTX), B16F10 melanoma, mesoporous silica nanoparticles, curcumin (CUR), D-α-tocopherol polyethylene glycol 1000 succinate (TPGS), P-glycoprotein (P-gp)

## Abstract

**Background/Objectives**: Paclitaxel (PTX) faces clinical limitations in melanoma treatment due to poor solubility, P-glycoprotein (P-gp)-mediated efflux, and systemic toxicity. This study aimed to develop PTX-loaded mesoporous silica nanoparticles (PS), which would be co-administered with curcumin (CUR) and D-α-tocopherol polyethylene glycol 1000 succinate (TPGS) to enhance intracellular accumulation and improve anti-tumor activity. CUR and TPGS were integrated with PS to inhibit P-gp-mediated PTX-efflux, to enhance the intracellular accumulation of PTX, and to improve anti-tumor activity in B16F10 cells. **Methods:** The physicochemical properties of PS were analyzed using standard characterization methods. The antitumor activity of PS co-administered with CUR and TPGS was evaluated using two-dimensional (2D) culture and three-dimensional (3D) spheroid assays, and also assessed in B16F10 tumor-bearing mice. The therapeutic mechanism of the PS combination was compared using apoptosis and microtubule disruption through flow cytometry and confocal microscopy. The pharmacokinetics and biodistribution of the PS combination were compared in B16F10 tumor-bearing mice. **Results:** PS formulations exhibited amorphous transformation with an approximate particle size of 200 nm. PS co-administered with CUR and TPGS reduced the IC_50_ to 178.7 nM compared with 283.3 nM for free PTX in B16F10 melanoma cells and achieved significant tumor growth inhibition in B16F10 melanoma spheroid culture. The intracellular accumulation of PTX correlated with its therapeutic efficacy. Flow cytometry revealed a significant induction of both early and late apoptosis in cells treated with the PS + CUR + TPGS combination, while confocal imaging confirmed enhanced microtubule disruption. In B16F10 tumor-bearing mice, PS co-administered with CUR and TPGS demonstrated higher and selective distribution of PTX into tumor tissue without affecting systemic exposure of PTX in B16F10-xenografted mice. **Conclusions:** PS + CUR + TPGS combination enhanced PTX delivery by improving solubility and enhancing distribution to tumor tissue through P-gp inhibition, thereby increasing its therapeutic potential. The combination of CUR and TPGS offers synergistic apoptosis induction and microtubule disruption. Thus, the PS + CUR + TPGS combination represents a promising approach for treating drug-resistant melanomas.

## 1. Introduction

Melanoma is the most lethal form of skin cancer, and the global occurrence of these cancers continues to increase. In the United States, approximately 100,640 new melanoma cases occurred in 2024, resulting in 8290 deaths. The 5-year relative survival rate of melanoma ranges significantly depending on the stage progression. In the early stage, when the cancer is localized, the survival rate exceeds 99%. However, the rate drops markedly to 35% following metastasis to a distant area [1].

Recent studies have highlighted that resistance to immune checkpoint inhibitors remains a substantial clinical hurdle in melanoma treatment [2]. Similarly, while targeted therapies have improved patient outcomes, the development of resistance mechanisms continues to impede their long-term efficacy. These findings underscore the necessity for ongoing research to overcome resistance and enhance the effectiveness of current melanoma therapies [3]. Against these backdrops, research into PTX-based formulations could offer promising opportunities for melanoma treatment. Notably, PTX, a widely used chemotherapeutic agent, has demonstrated efficacy across various cancer types, including melanoma [4,5]. Indeed, a phase III randomized controlled trial showed that nanoparticle albumin-bound paclitaxel (nab-PTX) significantly improved progression-free survival and disease control rates in chemotherapy-naive patients with metastatic melanoma compared to dacarbazine [6]. Additionally, research has indicated that ultralow, non-toxic doses of PTX can elicit a notable antitumor effect in vivo, as evidenced by prolonged survival in melanoma-bearing mice and a reduced tumor burden [7].

PTX administration is fundamentally limited by its extremely poor aqueous solubility (0.3 μg/mL), which necessitates the use of cremophor EL and ethanol for intravenous (IV) injection. However, cremophor EL is associated with a significant risk of severe hypersensitivity reactions, including anaphylaxis, dyspnea, hypotension, angioedema, and generalized urticaria [8]. Therefore, to mitigate these risks, patients must receive premedication with corticosteroids and antihistamines before each infusion [9]. Subcutaneous (SC) administration of PTX represents a transformative approach to overcoming the critical limitations of IV PTX injections [10]. By leveraging polymer-based prodrug formulations, such as polyacrylamide conjugates, SC delivery achieves a 70% reduction in peak plasma concentration (C_max_) compared to IV, while maintaining therapeutic efficacy and mitigating dose-limiting toxicities, including neutropenia [11]. That is, SC administration of the PTX formulation sustained tumor drug levels for >72 h post-injection, ensuring prolonged therapeutic exposure [11]. Moreover, SC formulations eliminate cremophor EL, thereby reducing systemic neurotoxicity and hypersensitivity reactions [12]. This administration route also simplifies treatment logistics, enabling potential self-administration and reducing hospital visits, which could significantly improve long-term patient compliance. Collectively, these pharmacokinetic and safety advantages position SC administration of PTX as a promising candidate for redefining the paradigms of outpatient cancer therapy.

Meanwhile, studies have indicated that PTX can inadvertently activate nuclear factor-kappa B (NF-κB), leading to the upregulation of matrix metalloproteinase-9 (MMP-9), which is associated with increased tumor invasiveness and metastasis. Specifically, PTX-induced NF-κB activation promotes the elevated expression of MMP-9, which contributes to the degradation of the extracellular matrix and facilitates cancer cell migration. This paradoxical effect imposes a significant limitation on PTX monotherapy, as it may promote cancer progression in certain contexts. Therefore, combining PTX with agents that inhibit NF-κB activation could enhance its therapeutic efficacy by mitigating these adverse effects [13].

Curcumin (CUR) is also a promising adjunctive agent to PTX, as it enhances the therapeutic efficacy of PTX and addresses some of the limitations associated with chemotherapy, including drug resistance and toxicity [14,15]. Furthermore, the ability of CUR to inhibit MMP-9 and upregulate the tissue inhibitor of metalloproteinases-2 (TIMP-2) significantly suppresses tumor angiogenesis, metastasis, and cell proliferation, thereby complementing the antitumor mechanisms of PTX [16,17]. In breast cancer cells (MCF-7), the combination of CUR and PTX has been shown to synergistically enhance growth inhibition and induce significant apoptosis [17]. Furthermore, CUR acts as a chemosensitizer by inhibiting the nuclear factor kappa B (NF-κB) pathway-mediated multidrug-resistance, thereby improving the cytotoxic activity of PTX, a substrate for P-glycoprotein (P-gp) [18]. Studies also demonstrated that CUR-loaded lipid nanoparticles inhibited P-gp-mediated drug efflux in multidrug-resistant cancer cells, restoring the susceptibility of cancer cells to PTX. Additionally, the incorporation of PTX and CUR into novel drug delivery systems, such as cationic PEGylated niosomes and solid lipid nanoparticles, enhances their bioavailability, cellular uptake, and sustained release, resulting in improved anticancer efficacy while minimizing toxicity to normal cells [18,19]. These findings underscore the potential of CUR-PTX combination therapy as an effective strategy for overcoming drug resistance and enhancing therapeutic outcomes in cancer treatment.

Tocopherol polyethylene glycol succinate (TPGS), a water-soluble derivative of vitamin E, serves as a solubilizing agent and has been extensively studied for its role in enhancing the delivery and efficacy of hydrophobic drugs such as PTX when incorporated into mesoporous silica nanoparticles [20]. Additionally, TPGS has been shown to inhibit P-gp, a key player in multidrug resistance, thereby increasing the intracellular concentration of P-gp substrates, including various chemotherapeutic drugs [21]. The amphiphilic nature of TPGS also facilitates enhanced cellular uptake of nanoparticles, promoting improved interactions with cancer cell membranes [22]. Furthermore, TPGS stabilizes CUR and promotes its sustained release, enhancing its synergistic anticancer properties alongside PTX, such as apoptosis induction and NF-κB inhibition [17,23]. While specific studies on TPGS in B16F10 melanoma models are limited, the general benefits in drug delivery and potential to enhance therapeutic efficacy make TPGS a promising component in PTX-based treatments for melanoma [24].

Therefore, this study aimed to develop a nano-based delivery system comprising PTX-loaded mesoporous silica nanoparticles for SC administration. Mesoporous silica nanoparticles have been approved by the United States Food and Drug Administration (U.S. FDA) for clinical trials of cancer formulations due to their adjustable porous structure, ability to induce surface modification, high loading efficiency, excellent biocompatibility, and biodegradability [25]. This study demonstrated that the insertion of PTX into mesoporous silica nanoparticles with co-administration of CUR and TPGS could efficiently deliver PTX to B16F10 by diminishing fatal problems associated with PTX in vivo and in vitro, yielding PSC (i.e., PS combined with CUR) and PSCT (i.e., PS combined with CUR and TPGS) formulations, respectively. These formulations are designed to enhance therapeutic efficacy by overcoming the inherent drawbacks associated with PTX through synergistic chemosensitization and modulation of P-gp activity.

## 2. Materials and Methods

### 2.1. Materials

Mesoporous silica nanoparticles, named Smart Mesoporous Ball 7 (SMB7; Patent no. KR-10-2240246) and PTX encapsulated SMB7 (PS), were provided by Dr. Sang-Cheol Han (CEN Co., Ltd.; Miryang-si, Republic of Korea). SMB7 contains SiO_2_ and ZnO (atomic composition: Si 30.4%, O 68.0%, and Zn 1.6%), while the average particle size of SMB7 was 200 nm, with pore diameter and volume of 2.54 nm and 0.81 cm^3^/g, respectively. PS was prepared by the solvent evaporation method. Briefly, 2 g of PTX was dissolved in a methanol: acetone = 1:1 (*v*/*v*) mixture in a round-bottom flask, then 8 g of SMB7 was added to the PTX solution and stirred for 1 h; the solvent was evaporated using a vacuum evaporator [26]. PS was purified through a multi-step washing process to remove unincorporated or surface-adsorbed PTX. Specifically, PS was dispersed in 100 mL of ethanol for 2 h, followed by centrifugation at 10,000× *g* for 10 min, and this process was repeated three times. Subsequently, the PS particles were dispersed in 100 mL of distilled water for 4 h and centrifuged at 10,000× *g* for 10 min, and this process was repeated twice to remove residual solvent and free drug molecules. The final nanoparticle product was recovered by centrifugation and dried under low vacuum conditions to obtain a dry powder suitable for biological experiments.

B16F10 cells were obtained from the Korean Cell Line Bank (Seoul, Republic of Korea). CUR, TPGS, berberine chloride (internal standard; IS), 3-(4,5-dimethylthiazol-2-yl)-2,5-diphenyltetrazolium bromide (MTT), dimethyl sulfoxide (DMSO), verapamil, PTX, bovine serum albumin (BSA), and formic acid were purchased from Sigma–Aldrich (St. Louis, MO, USA). Dulbecco’s Modified Eagle Medium (DMEM), phosphate-buffered solution (PBS), fetal bovine serum (FBS), and penicillin–streptomycin were purchased from Corning Life Sciences (Oneonta, NY, USA). Meanwhile, 4′,6-diamidino-2-phenylindole (DAPI) was purchased from Chem Cruz (Dallas, TX, USA). Goat anti-mouse IgG conjugated with FSD 594 was purchased from BioActs (Incheon, Republic of Korea). All other reagents were of cell culture grade or analytical grade.

### 2.2. Physicochemical Characteristics of PS

#### 2.2.1. Solubility

PTX loading in the mesoporous nanosilicate was measured as follows: 5 mg of the powder was placed in 50 mL of methanol and completely dissolved using a thermo micro-mixer (Finepcr; Gunpo-si, Republic of Korea) at 37 °C for 2 h. After 1 min of centrifugation, the supernatant was filtered through a 0.45 μm membrane filter. The filtrate was diluted 100-fold with the mobile phase and injected into the LC–MS/MS system to measure the PTX concentration. PTX loading (%) was calculated as the percentage of PTX amount in the total weight of the PS formulation.

To evaluate the solubility of the formulation, PTX and PS powder (2 mg as PTX) were weighed, and then, added in 2 mL of simulated body fluid (SBF; pH 7.4) consisting of 135 mM sodium chloride, 4.2 mM sodium bicarbonate, 4 mM potassium chloride, 1 mM potassium phosphate dibasic trihydrate, 1.5 mM magnesium chloride, 2.5 mM calcium chloride, 0.5 mM sodium sulfate 0.071 g, tris(hydroxymethyl)aminomethane 50 mM, and 40 mM hydrochloride. The mixtures were shaken using a thermo-micro mixer at 37 °C for 2 h. After 1 min of centrifugation, the supernatant was filtered through a 0.45 μm membrane filter. The filtrate was diluted 100-fold with the mobile phase and injected into the LC–MS/MS to measure the PTX concentration.

#### 2.2.2. Release Test

The in vitro drug release of PTX from the formulation was measured as follows: PTX and PS (all containing 2 mg PTX) were placed in the cellulose membrane pouch (Snakeskin^TM^ Dialysis Tubing 10,000 MWCO; Thermo Fisher Scientific Inc.; Waltham, MA, USA), and 5 mL of SBF (pH 7.4) was added inside the cellulose membrane pouch. The cellulose membrane pouch was put into 50 mL of SBF containing 0.5% Tween 80 in a 250 mL beaker and shaken at 100 rpm in a dual-motion shaker (Finepcr; Gunpo-si, Republic of Korea). Samples (100 μL aliquots) were collected for 48 h, and then diluted 100-fold with mobile phase and injected into the LC-MS/MS system to measure the PTX concentration.

Although the intrinsic solubility of PTX in aqueous media is low (0.3 µg/mL), SBF containing 0.5% Tween 80 used as a release medium significantly increases PTX solubility (i.e., PTX solubility > 10–100 µg/mL at 0.1–3% Tween 80) [27]. In this study, PS in 5 mL SBF was placed in a dialysis pouch, while the exterior release medium consisted of 50 mL of SBF + 0.5% Tween 80. This large external volume and surfactant content allowed the PTX released from the pouch to diffuse into the external medium and remain dissolved.

#### 2.2.3. Scanning Electron Microscopy (SEM)

The particle size and surface morphology of PTX, SMB7, and PS were observed using a SU8220 Scanning Electron Microscope (Hitachi; Tokyo, Japan). Samples were coated under conditions of 5.0 kV, 15 mA, and 10 min using a sputter coater (EMI Tech, K575k, West Sussex, UK).

#### 2.2.4. Differential Scanning Calorimetry (DSC)

The thermograms for PTX, SMB7, and PS were obtained using a thermal analysis instrument (DSC Q2000, V24.4 Build 116; TA Instruments, New Castle, DE, USA). Then, 5 mg of PTX, SMB7, or PS was placed in a Tzero aluminum pan. The instrument was operated under a nitrogen atmosphere (50.0 mL/min), and the following thermal program was applied: equilibration at 10 °C, followed by an isothermal step for 1 min, a temperature ramp of 5 °C/min up to 300 °C, and a final isothermal step for 1 min.

#### 2.2.5. X-Ray Diffraction (XRD)

An Empyrean X-ray diffractometer equipped with a copper anode (Malvern Panalytical Ltd.; Malvern, UK) was used to analyze the X-ray diffraction pattern of PTX, SMB7, and PS under the generator voltage of 40 kV and tube current of 30 mA. XRD patterns were recorded using Cu Kα radiation (λ = 1.54 Å) over a 2θ range of 5–70° with a scanning speed of 0.05°/s. Major diffraction peaks were identified and assigned Miller indices (hkl) based on matching observed 2θ values with literature-reported peaks for crystalline PTX.

#### 2.2.6. Fourier Transform Infrared Spectroscopy (FT-IR)

FT-IR pattern for PTX, SMB7, and PS was obtained using an FT-IR spectrophotometer (Frontier, PerkinElmer, Norwalk, CT, USA) with a scan range of 400–4000 cm^−1^

#### 2.2.7. Degradation of PS

Each 2 mg sample of SMB7 and PS was suspended in 1 mL of SBF (pH 7.4) and placed in a 2 mL tube. The tube was stirred in a Confido-S20H micro mixer incubator (Finepcr; Gunpo-si, Republic of Korea) at 37 °C at 700 rpm. The degradation profile of each formulation was monitored for 5 days. Every 24 h, a carbon-coated copper grid (Formvar/carbon 200 mesh; Ted Pella Inc.; Redding, CA, USA) was gently immersed in each 2 mL sample tube to allow the adsorption of the particles onto the grid surface. After approximately 1 min of soaking, the grid was removed, air-dried, and observed using an HT 7700 Bio-transmission electron microscope (Hitachi; Tokyo, Japan) to assess the structural integrity of the formulation over time.

### 2.3. Cell Viability

B16F10 cells were seeded at a density of 4 × 10^4^ cells per well in a 96-well plate and incubated at 37 °C in a 5% CO_2_ atmosphere for 24 h to allow cell attachment and stabilization before drug administration. After 24 h, the medium was replaced with fresh culture media containing various PTX formulations (i.e., PTX, PS, PS with CUR, PS with TPGS, and PS with verapamil) at increasing concentrations (0, 10, 20, 50, 100, 200, 500, and 1000 nM) of PTX or its equivalent amount.

After 48 h of drug exposure, MTT reagent (0.5 mg/mL in PBS, 15 μL) was added to each well, followed by an additional 4 h incubation to allow mitochondrial dehydrogenase enzymes in viable cells to reduce MTT into insoluble formazan crystals. The resulting formazan was then solubilized using 150 μL DMSO, and the absorbance was measured at 592 nm using an ultraviolet (UV) spectrophotometer. Cell viability was calculated relative to the untreated control group. Data were fitted using the inhibitory effect model $v=E_{max}(1-\frac{\left[I\right]}{{IC}_{50}+[I]})$. E_max_ and IC_50_ represent uninhibited cell viability (%) and the concentration of PTX (nM) at which half-maximal inhibition occurs. [I] represents the concentration of PTX.

### 2.4. Cellular Accumulation of PTX Formulation in B16F10 Cells

B16F10 cells were seeded at a density of 3 × 10^5^ cells per well in a 6-well plate and incubated at 37 °C with 5% CO_2_ overnight. The cells were treated with PTX formulations (i.e., PTX, PS, PS with CUR, PS with TPGS, and PS with verapamil) at 1 μM of PTX or its equivalent. After 1 h of incubation, cells were washed with PBS and harvested. Cells were added to 150 μL of IS solution (1 ng/mL berberine in 80% MeOH) and vortexed for 5 min, followed by centrifugation at 16,000× *g* and 4 °C for 5 min. A 120 μL aliquot of supernatant was transferred to an autosampler vial, and 1 μL was injected into the LC–MS/MS system to measure the intracellular PTX concentration.

### 2.5. Cytotoxicity of PTX Formulation in 3D Cultured B16F10 Spheroid

B16F10 cells were seeded at a density of 3 × 10^5^ cells per well in an ultra-low attachment 96-well plate to facilitate spheroid formation. Immediately after seeding, the plate was centrifuged at 15,000 rpm for 15 min to promote cell aggregation. Following centrifugation, the spheroids were allowed to stabilize for 24 h before drug treatment. The spheroids were treated with the following conditions and grown for 5 days: control, 200 μg/mL of SMB7, 200 μg/mL of curcumin, 25 μg/mL of PTX, PS (equivalent to 25 μg/mL PTX), PS with curcumin (equivalent to 25 μg/mL PTX + 200 μg/mL curcumin), and PS with TPGS (equivalent to 25 μg/mL PTX + 600 μg/mL TPGS).

During the culture period, the spheroid radius was measured daily to assess tumor growth inhibition across different treatment groups. After five days, the spheroids were harvested, disrupted into a single-cell suspension, and analyzed for intracellular PTX and curcumin concentrations using LC–MS/MS. Additionally, the culture medium was collected from each well and analyzed to quantify the remaining extracellular concentrations of PTX and curcumin, providing further insights into drug uptake and retention within the spheroids.

### 2.6. Apoptosis Analysis in B16F10 Cells Using Flow Cytometry

B16F10 cells were seeded at a density of 8 × 10^4^ cells in a cell culture dish and cultured until a confluency of 80% was reached. Upon reaching the desired confluency, the culture medium was aspirated and replaced with fresh medium containing one of the following treatments: control, PTX (25 μg/mL), PS (equivalent to 25 μg/mL PTX), PSC (equivalent to 25 μg/mL PTX + 200 μg/mL curcumin), and PSCT (equivalent to 25 μg/mL PTX + 200 μg/mL curcumin + 600 μg/mL TPGS).

After 24 h of treatment, the culture supernatant was collected into a 15 mL conical tube. Cells were then detached using 1.5 mL of trypsin, and the resulting cell suspension—including detached cells and debris—was combined with the previously collected medium in the same tube. The samples were centrifuged at 1000 rpm for 3 min, and the supernatant was carefully discarded. The cell pellet was resuspended in 1 mL of DMEM supplemented with 10% FBS and 1% penicillin–streptomycin. Subsequently, 100 μL of the Muse Annexin V and Dead Cell kit (Cytek Biosciences Inc., Fremont, CA, USA) was added to the cell suspension, and the samples were incubated at room temperature in the dark for 20 min. Apoptosis and cell viability were then assessed using the Guava^®^ Muse^®^ Cell Analyzer (Cytek Biosciences Inc., Fremont, CA, USA).

### 2.7. Confocal Staining

B16F10 cells were seeded at a density of 4 × 10^4^ cells per well in a 24-well plate and cultured until a confluency of 80% was reached. Once the desired confluency was achieved, the culture medium was replaced with fresh media containing the following respective treatments: control, 200 μg/mL of SMB7, 200 μg/mL of curcumin, 25 μg/mL of PTX, PS (equivalent to 25 μg/mL PTX), PS with curcumin (equivalent to 25 μg/mL PTX + 200 μg/mL curcumin), and PS with TPGS (equivalent to 25 μg/mL PTX + 600 μg/mL TPGS).

Cells were incubated post-treatment for 24 h and then fixed and stained to visualize intracellular structures. DAPI was used for DNA staining to highlight nuclear morphology, and goat anti-Mouse IgG conjugated with FSD 594 was employed for β-tubulin staining to observe microtubule organization. As PTX primarily acts by stabilizing microtubules, fluorescence images were acquired using an LSM700 confocal laser scanning microscope (Carl Zeiss GmbH, Oberkochen, Germany). Confocal images were acquired at 100 × magnification to enhance the visualization of DNA and β-tubulin structures, enabling the assessment of PTX-induced cytoskeletal disruption and apoptosis-related nuclear changes.

### 2.8. Antitumor Activity and Pharmacokinetic Study of PTX Formulation in B16F10 Xenograft Mice

C57BL/6 mice (male, 6 weeks old) were purchased from Samtaco (Osan, Republic of Korea) and used as B16F10 tumor xenograft models. Mice were maintained under pathogen-free conditions.

**Figure 1:**
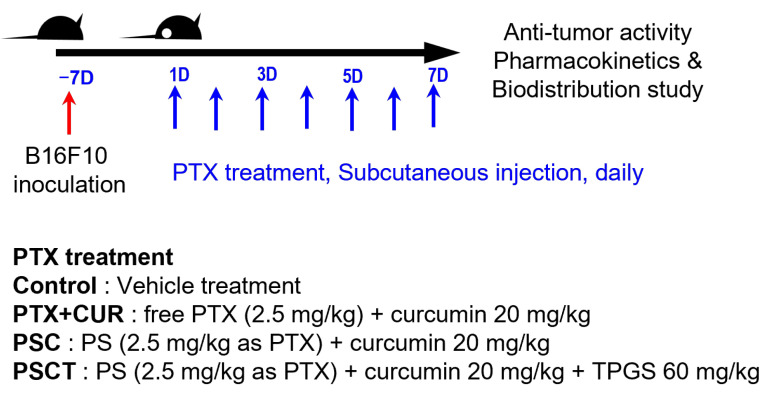
In vivo experimental design and dose scheduling in B16F10 tumor−bearing mice.

B16F10 melanoma cells (1 × 10^6^ cells in 200 µL PBS) were subcutaneously implanted into the dorsal flank of forty C57BL/6 mice. Tumor progression was monitored daily, and when the tumor volume reached approximately 100 mm^3^, each of the ten mice was randomly assigned to four treatment groups. Each group received SC injections of the assigned formulations daily for seven consecutive days (Figure 1). The minimum number of mice required to achieve adequate study power (significance level of 0.05 and statistical power of 80%) to monitor a 30% reduction in tumor volume change was estimated to be 10 mice per group based on our previous results of Kim et al. [26] and sample size calculators (clincalc.com). Based on these estimates, we conducted an antitumor activity study on a total of 40 B16F10 xenograft mice (10 mice per group).

During treatment, tumor volume and body weight were measured daily throughout this study using the following formula: *V* = 0.5 × *A* × *B*^2^. Here, A denotes length and B represents width. Mice were excluded if they showed signs of abnormal behavior, rapid tumor necrosis, tumor volume exceeding 3000 mm^3,^ or weight loss exceeding 20% during the study period. Two mice from the control groups died on the 5th and 6th days of vehicle treatment, and two mice were excluded because of tumor volume exceeding 3000 mm^3^ on the 7th day. Finally, six mice from the control group were included in the data analysis. No mice in the PTX + CUR, PSC, and PSCT groups did not meet the exclusion criteria during the experiment; thus, all animals were included in the final analysis, and pharmacokinetic and biodistribution analyses were performed.

After the final injection, the mice from PTX + CUR, PSC, and PSCT groups were randomly subdivided into two time points (*n* = 5 for each subgroup; 2 h and 8 h post-administration) for pharmacokinetic and biodistribution analyses. At the designated time points (0.25, 1, and 2 h from the 2 h group; 0.5, 4, and 8 h from the 8 h group), blood samples (approximately 80 µL) were collected from the retro-orbital plexus of mice. For the PTX and CUR analyses, 30 µL of plasma aliquots were stored at −80 °C. At 2 h, the mice were anesthetized, and the organs (heart, lung, liver, kidney, pancreas, and tumor) were harvested, weighed, and homogenized using a MM400 Laboratory Ball Mixer Mill (Retsch, Haan, Germany). Aliquots (50 µL) of 20% tissue homogenates prepared using 10% MeOH were stored at −80 °C for subsequent analyses.

Aliquots (30 μL) of plasma and aliquots (50 μL) of tissue homogenate samples were added with 120 and 150 μL of IS solution (1 ng/mL berberine in MeOH), respectively, and vortexed for 5 min, followed by 5 min centrifuged at 16,000× *g* at 4 °C. A 120 μL aliquot of supernatant was transferred to an autosampler vial and 1 μL was injected into the LC-MS/MS system.

### 2.9. SMB7 Toxicity Evaluation

Fourteen C57BL/6 mice (male, 6 weeks old) were randomly divided into two groups (*n* = 7 per group) and received daily subcutaneous injection either vehicle (saline 4 mL/kg) or SMB7 (100 mg/kg suspended in 4 mL saline) for seven consecutive days. After the completion of the treatment period, mice were humanely euthanized according to approved protocols. Major organs (heart, lung, kidney, pancreas, and liver) were harvested, gently blotted to remove excess blood, and weighed. Body weights were recorded daily throughout the experiment and at the final endpoint. The minimum number of mice required for adequate study power (significance level of 0.05 and statistical power of 80%) to monitor a 20% increase or decrease in organ weight change was estimated to be 3~7 mice per group for major organs based on our previous results of Kim et al. [26] and sample size calculators (clincalc.com). Based on these estimates, we conducted a toxicity assessment on a total of 14 mice (7 mice per group).

### 2.10. LC-MS/MS Analysis

The concentrations of PTX and CUR in the biological samples were analyzed simultaneously using a TSQ Altis Plus LC-MS/MS system (Thermo Fisher Scientific Inc.; Waltham, MA, USA). The separation was performed on a Luna C18 column (2.0 × 150 mm, 5 μm particle size; Phenomenex, Torrance, CA, USA) using a mobile phase consisting of water and methanol (20:80, *v*/*v*) with 0.1% formic acid at a flow rate of 0.2 mL/min. The column temperature was maintained at 40 °C, and the injection volume was 1 μL. Quantification was performed using multiple reaction monitoring (MRM) at m/z 876.4 → 308.0 for PTX (retention time T_R_ 3.09 min), at m/z 369.1 → 285.0 for CUR (T_R_ 3.16 min), and at m/z 336.2 → 320.1 for berberine (IS; T_R_ 2.34 min). The fragmentor voltage was 25 V, and collision energies for 16–27 eV for PTX, CUR, and berberine in a positive ionization mode.

### 2.11. Data Analysis

Non-compartmental methods (WinNonlin version 5.1, Pharsight Co., Mountain View, CA, USA) were used to estimate the pharmacokinetic parameters.

The biodistribution of PTX in various mouse tissues was quantified and expressed as a percentage of the injected dose per gram of tissue (%ID/g). This parameter reflects the proportion of the administered drug accumulated in a specific organ, normalized by the weight of the organ. The following formula was used to calculate the drug concentration in the tissue, which is measured (e.g., ng), normalized to the total injected dose, and further corrected based on the weight of the harvested tissue to standardize drug accumulation across different organs as follows: $\%ID/g=\left(\frac{Drug\;amount\;in\;each\;organ\left(mg\right)}{Injected\;Dose\;\left(mg\right)}\right)\times 100\times \frac{1}{Organ\;weight\;(g)}$.

An analysis of variance (ANOVA) and student’s *t*-test were used to analyze statistical significance using SPSS Statistics for Windows, version 27 (IBM Corp., Armonk, NY, USA).

## 3. Results

### 3.1. Physicochemical Characterization of PS Formulation

**Figure 2:**
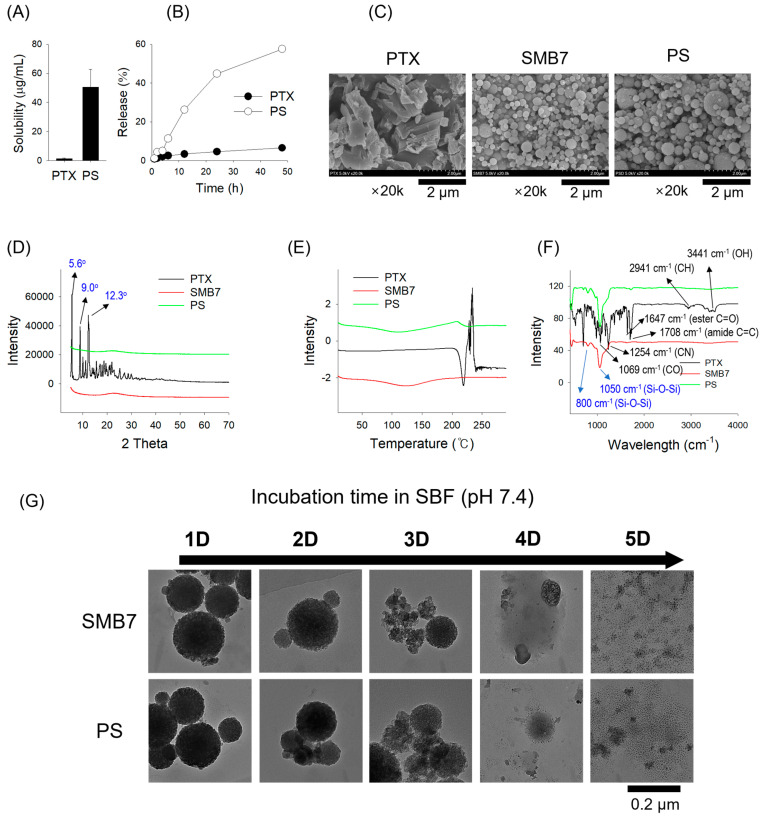
Physicochemical characteristics of PTX, SMB7, and PTX-loaded SMB7 (PS) using various analytical techniques. (**A**) Solubility and (**B**) release pattern of PTX and PS (2 mg PTX and its equivalent). Data are presented as the mean ± SD (*n* = 3). (**C**) Scanning electron microscopy (SEM) images, (**D**) X-ray diffraction (XRD) patterns, (**E**) differential scanning calorimetry (DSC) thermograms, and (**F**) Fourier transform infrared spectroscopy (FT-IR) spectra of PTX, SMB7, and PS (10 mg each). Characteristic peak values for PTX and SMB7 are marked in black and blue, respectively. (**G**) Degradation profiles of SMB7 and PS (2 mg each) in simulated body fluid (SBF) over 5 days.

The PTX-loading in the PS formulation was determined as 24.4 ± 3.78% and the PTX solubility in PS was increased 35.3-fold (Figure 2A). PTX showed a very poor drug release at 6.58% over 48 h. However, the PS formulation showed a significant increase to 57.6% for 48 h (Figure 2B). These results suggest that incorporating PTX into mesoporous nanosilicate can significantly increase the solubility and release profile of PTX compared with free PTX. SEM images were employed to evaluate the surface morphologies of PTX, SMB7, and PS (Figure 2C). PTX exhibited various crystal shapes, such as rectangular or rod-like structures, while SMB7 presented spherical shapes. PS also exhibited the same spherical shape as SMB7, with no visible PTX particles, thereby indicating successful drug encapsulation without altering the particle structure (Figure 2C).

The XRD pattern of pure PTX exhibited three well-defined crystalline peaks at approximately 2θ of 5.6°, 9.0°, and 12.3°, with calculated d-spacings of 15.9 Å, 9.8 Å, and 7.2 Å, respectively. These peaks can be assigned to Miller indices of (100), (110)/(200), and (210), in close agreement with reported patterns for free PTX [28]. The presence of these sharp diffraction peaks confirms that PTX remains in its crystalline form within the sample. In contrast to the PTX sample, both SMB7 and PS XRD patterns displayed a broad halo pattern without distinct sharp peaks and demonstrated a similar pattern to SMB7 (Figure 2D). This is indicative of amorphous mesoporous silica structures, consistent with previous studies reporting broad diffraction features in amorphous silica matrices at around 2θ of 2–3° or ~22° [29]. The disappearance of the characteristic crystalline peaks of PTX along with a similar pattern to SMB7 in the PS formulation suggested that PTX underwent a structural transition from a crystalline to an amorphous form upon incorporation into the SMB7 matrix. The DSC thermogram (Figure 2E) confirmed the XRD result. Pure PTX showed a distinct endothermic peak at 220 °C, corresponding to its melting point, confirming its crystalline nature. In contrast, PS exhibited a complete absence of this melting peak, with a broad thermal baseline shift around 120 °C, suggesting a glass transition temperature (Tg) typical of amorphous materials. This shift indicates the loss of crystalline order and the presence of an amorphous phase.

The FT-IR spectra were analyzed to assess drug–silica interactions in the solid state (Figure 2F). Pure PTX exhibited characteristic absorption bands at 3441 cm^−1^ (O–H stretching), 3309 cm^−1^ (N–H stretching), 2920–2850 cm^−1^ (aromatic C–H stretching), 1708 cm^−1^ (ester C=O stretching), 1647 cm^−1^ (amide C=C stretching), 1254 cm^−1^ (C–N stretching), and 1069 cm^−1^ (C–O stretching) sharply visible in its spectrum [30]. In contrast, in the FT-IR spectra of SMB7 and PS, these PTX-specific peaks are absent, while the dominant features correspond to silica, such as broad Si–O–Si stretching bands centered at 1050 cm^−1^ and 800 cm^−1^ [31]. The disappearance of PTX crystalline signals and the persistence of silica bands in FT-IR spectra of PS particles suggest that PTX is molecularly dispersed within the SMB7 matrix in an amorphous state, rather than forming distinct crystalline domains, and PTX was predominantly and physically entrapped within the SMB7 matrix. The results were consistent with previous reports on paclitaxel-loaded silica systems [32].

Both SMB7 and PS exhibited a gradual degradation profile over the 5-day incubation period in SBF (Figure 2G). While the spherical morphology was maintained until day 2, significant structural collapse was observed from day 3, with nearly complete disintegration by day 5. Notably, PS showed a degradation pattern comparable to that of bare SMB7, indicating that the loading of paclitaxel did not affect the structural stability of the carrier.

### 3.2. Effect of PTX and PS on the Cell Viability and Spheroid Growth of B16F10 Melanoma Cells

Next, an MTT assay was conducted on B16F10 melanoma cells to assess the cytotoxic effects of PTX formulations and evaluate the impact of P-gp inhibition. This study included formulations with PTX alone, PS, and PS combinations with known P-gp inhibitors such as CUR and TPGS, while verapamil, a well-established P-gp inhibitor, was also included as a positive control to benchmark the inhibitory potential of CUR and TPGS.

**Figure 3:**
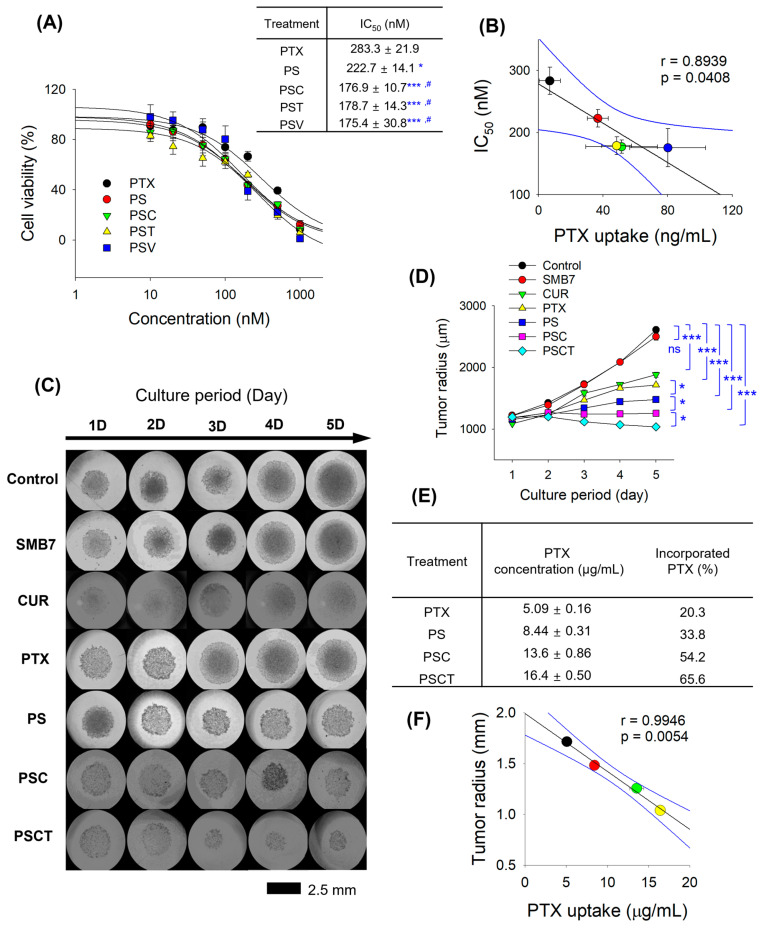
In vitro anticancer effects of PTX and PS in B16F10 melanoma cells. (**A**) The effect of PTX and PS with a combination of CUR, TPGS, and verapamil (i.e., PSC, PST, and PSV, respectively) on the viability of B16F10 cells was measured in the concentration range of 0–1000 nM PTX. IC_50_ values of PTX were calculated from the cell viability results. * *p* < 0.05, *** *p* < 0.001 compared with control group; # *p* < 0.05 compared with PS group. (**B**) Correlation between PTX uptake and IC_50_ values of various treatments in B16F10 cells. For the PTX uptake experiment, cells were treated with 1 µM PTX or its equivalent formulations for 1 h. (**C**) Representative images and (**D**) spheroid radius of B16F10 spheroids following the treatment of SMB7 (200 µg/mL), CUR (200 µg/mL), PTX (25 µg/mL), and PS with a combination of CUR and TPGS [i.e., PSC (equivalent to 25 μg/mL PTX + 200 μg/mL CUR), PSCT (equivalent to 25 μg/mL PTX + 200 μg/mL CUR + 600 μg/mL TPGS), respectively] for 5 days. (**E**) Intracellular accumulation of PTX in B16F10 spheroids after 5 days of treatment with PTX, PS, PSC, and PSCT. (**F**) Correlation between PTX uptake and tumor radius of B16F10 spheroids after 5 days of treatment with PTX, PS, PSC, and PSCT. Data are presented as the mean ± SD (*n* = 4).

The dose–response curves demonstrated a significant shift in IC_50_ values among different formulations (Figure 3A), indicating varying levels of cytotoxic efficacy by PTX formulation combined with P-gp inhibitors. That is, the improvement in cytotoxicity was about 21.4%. Further addition of CUR, TPGS, and verapamil resulted in a cytotoxicity enhancement of 36.9–38.1% compared with the PTX group and significantly reduced IC_50_ values compared with the PS group. We also monitored the cellular accumulation of PTX in the presence or absence of PS or P-gp inhibitors. Combinations of CUR, TPGS, and verapamil with PS exhibited additional P-gp inhibition and, thereby, increased PTX accumulation into B16F10 cells compared to the PTX or PS group. Therefore, PTX accumulation was increased by the presence of PS formulation and P-gp inhibitors, and it was correlated with IC_50_ values of PTX combination in B16F10 cells (Figure 3B). The results suggested that a higher PTX concentration in B16F10 cells would result in a more effective anticancer effect, and the concomitant administration of a P-gp inhibitor could be a more effective therapeutic strategy to enhance the antitumor efficacy of PTX.

To evaluate the anticancer efficacy of PTX-loaded formulations under physiologically relevant conditions, the tumor growth inhibition by treating PTX formulations with CUR and TPGS was investigated using B16F10 spheroids over five days. In the control group, the spheroid diameter increased over the 5-day culture period, and the addition of SMB7 did not interfere with the tumor growth of B16F10 spheroids. CUR and PTX significantly suppressed the tumor growth of B16F10 spheroids. PS treatment significantly decreased the radius of B16F10 spheroids compared with the PTX group, which was consistent with the reduced cytotoxic effect of PS observed in the MTT assay results for B16F10 cells. The combination of CUR and PS (i.e., PSC group) further suppressed the tumor growth of B16F10 spheroids, and PSCT treatment exhibited the most significant suppression of spheroid growth, as confirmed by tumor radius measurements (Figure 3C,D). Statistical analysis revealed a significant difference (*p* < 0.05) between the PSCT and PSC groups as well as between the PSC and PS groups, highlighting the superiority of the combination of PS, CUR, and TPGS. Additionally, intracellular PTX quantification revealed that PSCT had the highest PTX retention (65.6% of the PTX was incorporated), followed by PSC (54.2%), PS (33.8%), and free PTX (20.3%) (Figure 3E). These data suggest that PS formulation significantly enhances PTX penetration and retention within tumor spheroids, and also supports enhanced antitumor activity through PTX-loaded nanosilicate and a combination of CUR and TPGS. Moreover, PTX accumulation in B16F10 spheroids was correlated with the tumor radius after the corresponding treatment (Figure 3F). These results suggest that a higher PTX concentration in B16F10 cells would enhance the anticancer effect, with a higher correlation coefficient than that observed in the MTT assay.

### 3.3. Apoptosis Analysis After the Treatment of PS with CUR and TPGS

**Figure 4:**
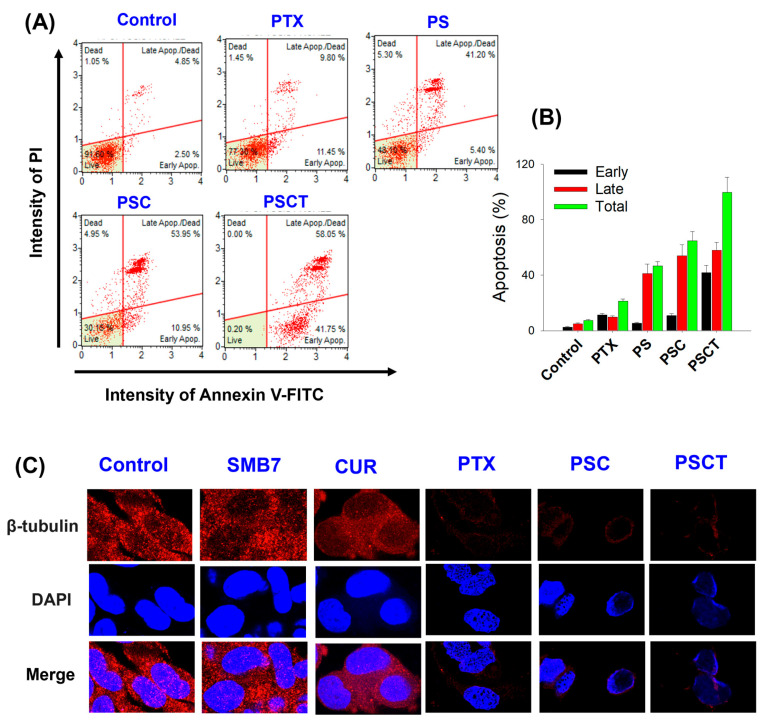
(**A**) Flow cytometry analysis of B16F10 cells. Annexin V-FITC/PI staining, showing the distribution of live, early apoptotic, late apoptotic, and dead cells after treatment with control, PTX (25 µg/mL), PS (equivalent to 25 μg/mL PTX), PSC (equivalent to 25 μg/mL PTX + 200 μg/mL CUR), and PSCT (equivalent to 25 μg/mL PTX + 200 μg/mL CUR + 600 μg/mL TPGS) for 24 h. (**B**) Percentage of early apoptosis, late apoptosis, and total apoptosis in B16F10 cells by different treatment groups, as determined by flow cytometry. (**C**) Confocal microscopy images of B16F10 cells stained for β-tubulin (red) and nuclei (DAPI; blue) after treatment with control, SMB7 (200 µg/mL), CUR (200 µg/mL), PTX (25 µg/mL), PSC (equivalent to 25 μg/mL PTX + 200 μg/mL CUR), and PSCT (equivalent to 25 μg/mL PTX + 200 μg/mL CUR + 600 μg/mL TPGS) for 24 h, illustrating microtubule integrity and nuclear morphology under different conditions. Confocal images were acquired at 100× magnification.

Flow cytometry analysis was performed using Annexin V-FITC and PI staining to evaluate the apoptotic effects of PTX formulations further (Figure 4A). This analysis enabled the differentiation between early apoptotic, late apoptotic, and necrotic cell populations. The percentage of apoptotic cells was quantified across different treatment groups, including control, PTX, PS, PSC, and PSCT. Flow cytometry dot plots demonstrated that a late apoptotic signal emerged following PTX treatment, accompanied by a significant increase in late apoptosis in cells treated with PS, PSC, and PSCT, as compared to PTX treatment (Figure 4A). When expressed as a percentage of apoptosis, early apoptosis was measured at 2.5%, while late apoptosis accounted for 4.8%, resulting in a total apoptosis rate of 7.2% in the control group. Cells treated with PTX exhibited an early apoptosis rate of 11.5% and a late apoptosis rate of 9.8%, leading to a total apoptotic rate of 21.3%. In the PS-treated group, early apoptosis was observed at 5.4%, while late apoptosis reached 41.2%, resulting in an overall apoptotic rate of 46.6%. For the PSC group, early apoptosis was measured at 11.0%, with late apoptosis increasing to 54.0%, presenting a total apoptosis rate of 65.0%. Cells treated with PSCT exhibited the highest induction of apoptosis, with early apoptosis accounting for 41.9% and late apoptosis reaching 58.1%, leading to a total apoptosis rate of 100% (Figure 4B). These results indicate that PSCT induced the highest apoptotic rate (100%), surpassing all other formulations, suggesting enhanced cytotoxic efficiency. Compared to free PTX (21.3%), PSCT exhibited a more than 4-fold increase in apoptosis induction, highlighting the role of TPGS and CUR in synergistically enhancing the apoptotic signaling.

### 3.4. Confocal Microscopy Analysis After the Treatment of PS with CUR and TPGS

To further assess the efficacy of the PTX formulations at the cellular level, confocal microscopy analysis was performed using B16F10 melanoma cells treated with control, SMB7, CUR, PTX, PSC, and PSCT (Figure 4C). Cells treated with the control and SMB7 exhibited strong β-tubulin expression, indicating intact microtubule integrity and no observable cytotoxic effects. Comparatively, cells treated with CUR showed a moderate reduction in β-tubulin expression, suggesting partial disruption of microtubule stability, which is consistent with a previous report [33]. This was accompanied by mild nuclear condensation, indicating early apoptotic events.

PTX-treated cells exhibited a pronounced decrease in β-tubulin intensity, indicative of microtubule disruption. Additionally, nuclear fragmentation was observed, confirming PTX-induced apoptosis through mitotic arrest. Cells treated with PSC displayed further suppression of β-tubulin expression, indicating enhanced disruption of microtubule networks. This was accompanied by pronounced nuclear condensation, suggesting an increase in apoptotic activity compared to PTX alone. The most pronounced reduction in β-tubulin expression was observed in cells treated with PSCT, indicating maximal disruption of microtubules. Extensive nuclear fragmentation and chromatin condensation were also evident, suggesting that CUR and TPGS enhanced PTX retention and potentiated apoptosis (Figure 4C).

### 3.5. Anticancer Activity of PS with CUR and TPGS in B16F10 Melanoma-Bearing Mice

**Figure 5:**
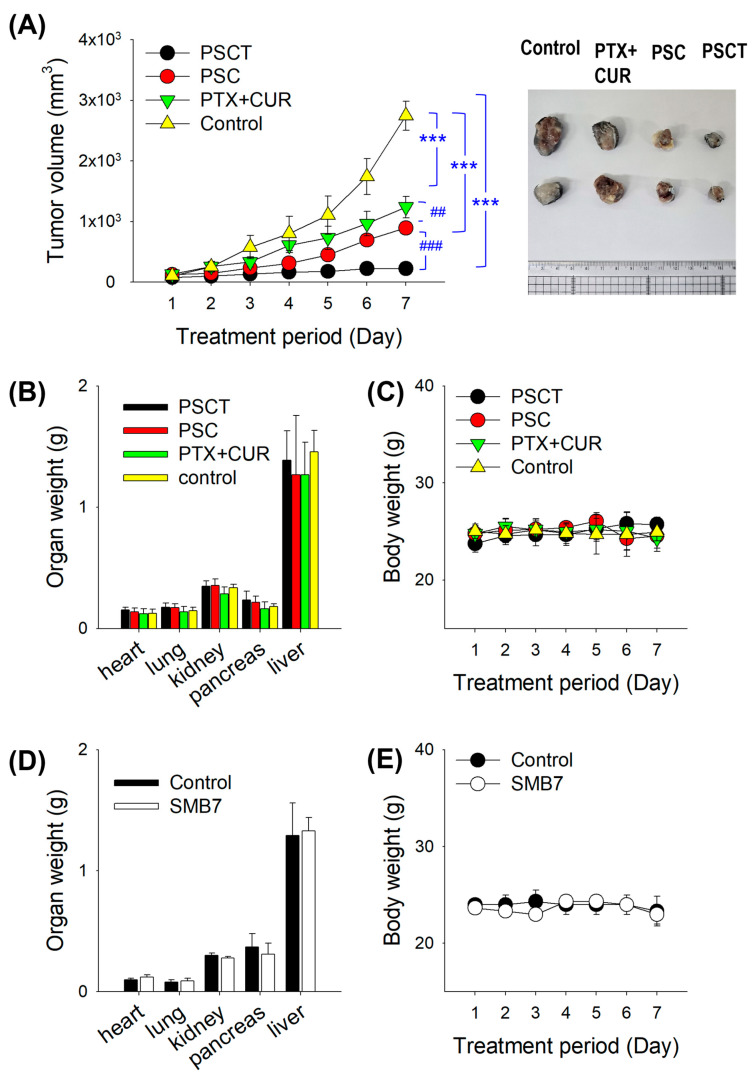
(**A**) Tumor growth trend in B16F10 melanoma-bearing mice following repeated SC administration of PTX formulations (i.e., control, PTX + CUR, PSC, and PSCT groups). *** *p* < 0.001 compared with control group; ## *p* < 0.01, ### *p* < 0.001 compared with PSC group. Data represent mean ± SD (*n* = 6–10). The right panel shows representative tumor tissues from the control, PTX + CUR, PSC, and PSCT groups. (**B**) Body weight and (**C**) organ weights of B16F10 melanoma-bearing mice after treatment with PTX formulations. B16F10 melanoma-bearing mice received daily subcutaneous injections for seven consecutive days with vehicle (saline 4 mL/kg), PTX + CUR (2.5 mg/kg PTX + 20 mg/kg CUR in 4 mL saline), PSC (equivalent to 2.5 mg/kg PTX + 20 mg/kg CUR in 4 mL saline), and PSCT (equivalent to 2.5 mg/kg PTX + 20 mg/kg CUR + 60 mg/kg TPGS in 4 mL saline). Data represent mean ± SD (*n* = 6–10). (**D**) Body weight and (**E**) organ weights of mice following repeated SC administration (daily for seven consecutive days) of vehicle (saline 4 mL/kg) and SMB7 (100 mg/kg in 4 mL saline). Data represent mean ± SD (*n* = 7).

To investigate the anticancer activity of the PTX formulation in vivo, B16F10 melanoma-bearing mice were subcutaneously injected according to the dosing schedule outlined in Figure 1. The tumor volumes in the control mice increased over time. Concomitant administration of free PTX with CUR significantly reduced tumor volume compared to the control group. Co-administration of PS and CUR (i.e., PSC) further inhibited tumor growth compared to the free PTX group with CUR. Co-administration of PS with CUR and TPGS (i.e., PSCT) significantly reduced tumor volume compared to the PSC group (Figure 5A). Meanwhile, none of these treatments caused a significant difference in body weight and organ weight (Figure 5B,C), suggesting no significant systemic toxicity during the PTX formulation treatment.

We then assessed the toxicity of SMB7 in mice. Seven days of SC administration of SMB7 did not affect the body weight and organ weight in the non-allograft model (Figure 5D,E). Moreover, there was no difference in the morphological appearance of organs between the control and SMB7-treated groups. These results suggest that SMB7 itself did not cause significant systemic toxicity and, therefore, improved the therapeutic index of PTX using PS formulation without inducing systemic toxicity during the one-week treatment period.

### 3.6. Pharmacokinetics and Biodistribution of PS with CUR and TPGS in B16F10 Melanoma-Bearing Mice

Next, plasma concentrations of PTX were measured following the final administration of PTX, as per the PTX dosing schedule, to investigate the steady-state pharmacokinetics and biodistribution of PTX after treatment with PS combined with CUR and TPGS (Figure 1).

**Figure 6:**
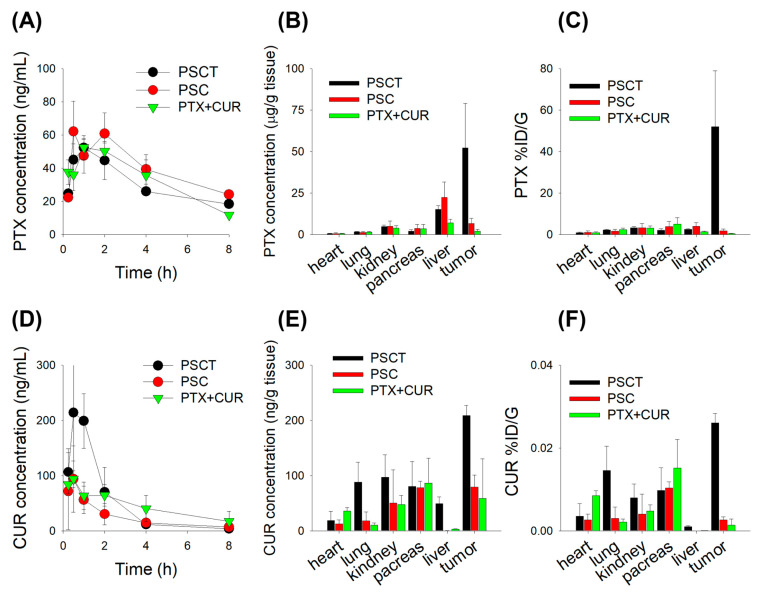
(**A**) Plasma PTX concentration–time profiles in B16F10 melanoma-bearing mice following subcutaneous administration of PTX formulations. B16F10 melanoma-bearing mice received daily subcutaneous injections for seven consecutive days with PTX + CUR (2.5 mg/kg PTX + 20 mg/kg CUR in 4 mL saline), PSC (equivalent to 2.5 mg/kg PTX + 20 mg/kg CUR in 4 mL saline), and PSCT (equivalent to 2.5 mg/kg PTX + 20 mg/kg CUR + 60 mg/kg TPGS in 4 mL saline). (**B**) PTX concentrations in major organs and tumor tissues at 2 h after administration of PTX formulations. (**C**) PTX distribution (%ID/g) in organs and tumor at 2 h after administration of PTX formulations. (**D**) Plasma CUR concentration–time profiles following administration of PTX formulations. (**E**) CUR concentrations in major organs and tumor tissues at 2 h after administration of PTX formulations. (**F**) CUR distribution (%ID/g) in organs and tumor at 2 h after administration of PTX formulations. Data represent the mean ± SD (*n* = 5).

Plasma concentration-time curves for PTX + CUR, PSC, and PSCT showed similar profiles and pharmacokinetic parameters (Figure 6A; Table 1). However, the organ distribution demonstrated that PTX was distributed to major organs, showing more than a 100-fold tissue concentration in the heart, lung, kidney, pancreas, liver, and tumor tissues. Meanwhile, the distribution of PTX to the liver and tumor tissue was significantly enhanced following PSC treatment. Indeed, PSCT treatment exhibited the highest PTX exposure in tumor tissue without excessive plasma accumulation (Figure 6B). When PTX biodistribution was expressed as %ID/g (percentage of the injected dose per gram of tissue), PTX showed a similar biodistribution pattern among the measured organs but exhibited a markedly increased uptake in tumor tissue following the administered combination of PS with CUR and TPGS (i.e., PSCT) (Figure 6C).

Additionally, CUR PK parameters were analyzed to assess their systemic exposure and impact on PTX formulations. These results showed that PSCT exhibited the highest curcumin exposure along with the presence of TPGS, whereas PTX and the PS formulation did not alter the CUR plasma profiles, suggesting improved CUR bioavailability in the PSCT treatment group (Figure 6D; Table 1). In contrast to PTX, the tissue concentration of CUR in the heart, lung, kidney, pancreas, liver, and tumor tissue was lower than the C_max_, indicating limited tissue distribution of CUR. In contrast, the CUR distribution to lung, kidney, liver, and tumor tissue was greatly enhanced after applying the PSCT treatment, which may be attributed to the contribution of TPGS in addition to the increased plasma exposure of CUR in the PSCT group (Figure 6E). When CUR biodistribution is expressed as %ID/g, the CUR showed much lower biodistribution to major organs than PTX and exhibited a significant increase in lung and tumor tissue after using the PS formulation in combination with CUR and TPGS (i.e., PSCT) (Figure 6F). TPGS, known for its P-gp inhibition and bioavailability-enhancing properties, is likely to contribute to the increased tumor retention of PTX and CUR by inhibiting P-gp-mediated efflux and, consequently, improve the therapeutic index of PTX in the PSCT treatment group.

## 4. Discussion

After approval by the US FDA as a medication in 1993, PTX has been established as a first-line therapy for ovarian, breast, lung cancers, and melanoma, demonstrating excellent efficacy, especially when used in combination with other chemotherapeutic agents [34]. PTX functions as a mitotic inhibitor by disrupting microtubule spindle assembly, thereby inhibiting chromosome segregation and cell division. However, poor solubility and low tumor accumulation of PTX due to P-gp-mediated efflux have limited its clinical application [35]. To overcome these limitations and enhance solubility while inhibiting P-gp, we introduced PTX-loaded mesoporous silica nanoparticles, co-administered with CUR and TPGS.

In our previous study, CUR was fully embedded within mesoporous nanosilicate SMB7, as confirmed through SEM and FT-IR analyses. Additionally, data from XRD and DSC revealed that the physicochemical properties of CUR-loaded SMB7 promoted an amorphous state [26]. Similarly, the irregular square-shaped crystalline structures of PTX were not observed in the PS formulation during SEM analysis. Considering the broad halo pattern in XRD, the absence of a distinct melting peak in DSCs with a thermal shift from approximately 220 °C to ~100 °C, and the broadening of FT-IR peaks, it can be concluded that PS exists predominantly in an amorphous state. Moreover, the PS formulation exhibited natural biodegradation, initiating within 2 days and achieving complete degradation within 5 days (Figure 2). A previous study investigated the IC_50_ of SMB7 on B16F10 cells and found that SMB7 did not cause cytotoxicity [26]. Additionally, our research, including both in vitro cytotoxicity assays and an in vivo study on SMB7 cytotoxicity, confirmed no observed toxicity in biological systems (Figure 5D,E).

Next, we investigate the in vitro anticancer effect of PTX, PS, PSC, and PSCT in melanoma cells (Figure 3). Unlike traditional 2D monolayer cultures, 3D spheroid cytotoxicity assays provide a more physiologically relevant model that closely mimics the tumor microenvironment [36]. In 2D cultures, the absence of an extracellular matrix limits cell–cell and cell–matrix interactions, leading to simplified drug responses and exaggerated sensitivity. Conversely, 3D spheroids enable the formation of extracellular matrix-like structures, promoting natural cell adhesion, signaling pathways, and the development of nutrient and oxygen gradients. This architecture creates drug penetration barriers, contributing to enhanced resistance mechanisms that reflect in vivo conditions [37]. Additionally, B16F10 melanoma spheroid models capture tumor heterogeneity, enabling the evaluation of long-term drug efficacy and apoptotic responses [38]. Thus, B16F10 spheroid assays offer superior predictive value for anticancer drug performance compared to conventional 2D assays. This was evidenced by the higher correlation coefficient obtained from B16F10 spheroids than from conventional 2D assays (Figure 3B,F). In addition, the elevated PTX accumulation in PSCT-treated spheroids suggests that CUR and TPGS effectively inhibit the P-gp-mediated efflux, resulting in sustained drug retention and prolonged cytotoxic effects. Moreover, the enhanced permeation and retention (EPR) effect facilitates the entry of nanosized silicate nanoparticles into the tumor microenvironment, as well as their cellular accumulation [39].

The improved cytotoxicity and enhanced intracellular accumulation observed in the PSCT-treated B16F10 2D cultures and 3D spheroids suggest that CUR and TPGS not only enhanced the intracellular accumulation of PTX by inhibiting P-gp activity but also facilitated mitochondrial dysfunction, leading to apoptosis (Figure 4). The combined data from 3D spheroid cytotoxicity, MTT assays, and PTX accumulation studies highlight the therapeutic potential of PSCT as an optimized drug delivery system, offering improved drug retention, enhanced cytotoxicity, and superior tumor growth inhibition compared to conventional PTX formulations. The interplay between PTX uptake, microtubule disruption, and apoptosis induction was demonstrated through uptake studies, confocal imaging, and flow cytometry (Figure 3E and Figure 4).

PTX functions as a mitotic inhibitor by disrupting microtubule spindle assembly, thereby inhibiting chromosome segregation and cell division. This mechanism leads to G2/M phase arrest and triggers apoptosis [35]. This process is tightly linked to the generation of reactive oxygen species and the activation of caspase cascades, which ultimately lead to apoptosis [40]. It is known that PTX-induced microtubule dysfunction initiates apoptosis via both c-Jun N-terminal kinase (JNK)-dependent and -independent pathways in many cancer cells, leading to the activation of caspase-3 and PARP cleavage, which are key markers of the apoptotic process [41,42]. In this study, the downstream effect of microtubule disruption by PTX was reflected in the flow cytometry apoptosis analysis. While late apoptosis percentages were similar across PS, PSC, and PSCT, early apoptosis showed substantial differences. The early apoptosis rate was increased from ~5.4% in PS to ~11.0% in PSC, highlighting the role of CUR as a P-gp inhibitor that enhances intracellular drug retention. Notably, PSCT, which includes both CUR and TPGS, exhibited an early apoptosis rate of ~41.75%, nearly 4-fold higher than PSC (Figure 4A,B).

CUR, while known for its P-gp inhibitory properties, also interferes with tubulin dynamics. CUR binds to tubulin, disrupts microtubule polymerization, reduces GTPase activity, and promotes tubulin dimer aggregation, leading to cell cycle arrest at the G2/M phase and subsequent apoptosis [43]. Confocal imaging revealed that CUR reduces microtubule network density compared to the control and SMB7, although not to the extent observed with PTX. PSC demonstrated enhanced anticancer effects, while the addition of TPGS further amplified these effects due to its strong P-gp inhibition (Figure 4C).

TPGS directly binds to the ATP-binding site of P-gp, inhibiting ATPase activity and inducing structural changes that impair drug efflux function [21]. In contrast, CUR indirectly suppresses ATPase activity and reduces P-gp expression through the PI3K/Akt/NF-κB signaling pathway [44]. Due to these differences, TPGS is more effective in directly inhibiting P-gp function, while CUR excels in regulating expression and suppressing energy metabolism. Thus, utilizing these distinct mechanisms may provide a synergistic effect in P-gp inhibition. The combined results from confocal microscopy and uptake assays confirm the dual role of CUR in inhibiting tubulin polymerization and P-gp activity; meanwhile, TPGS primarily enhances drug accumulation by inhibiting P-gp. This suggests a synergistic effect of CUR and TPGS in inhibiting P-gp, resulting in sustained PTX accumulation and the rapid, early activation of apoptotic signaling. Moreover, the combination of CUR and TPGS likely enhances mitochondrial membrane destabilization, thereby further promoting the initiation of apoptosis [45,46]. In summary, improved PTX uptake via P-gp inhibition (by CUR and TPGS) leads to greater microtubule disruption, ultimately accelerating early apoptosis. This mechanistic pathway underscores the potential of the PSCT formulation to overcome drug resistance and improve anticancer efficacy through optimized drug delivery and apoptotic induction.

Similar previous studies have reported enhanced tumor accumulation and efficacy using PTX-loaded mesoporous silica carriers. Fu et al. demonstrated a 6.5-fold increase in tumor PTX accumulation and a 3.2-fold extended peritoneal residence time compared to free PTX [47]. Our PSCT system exhibited a 135-fold higher %ID/g compared with free PTX + CUR and a 20.8-fold tumor-to-liver PTX concentration ratio. Considering the %ID/g metric offers critical insight into the selectivity and efficiency of tumor targeting, PSCT treatment achieved markedly higher tumor-specific accumulation of PTX compared to all treatment groups, highlighting its superior delivery efficiency and specificity (Figure 6C). This is particularly significant when considering off-target organ accumulation, especially in the liver, a common site of clearance for nanoparticles. The tumor-to-liver PTX ratio was highest in PSCT, indicating a favorable biodistribution profile and minimized hepatic burden (Figure 6C).

Regarding the PTX release profile, Jia et al. reported that spherical mesoporous silica nanoparticles with a small pore diameter (3.03 nm) yielded sustained PTX release and showed higher AUC and longer MRT values compared to larger pore size (9.68 nm) [48]. Additionally, Fang et al. demonstrated that PTX-loaded mesoporous silica nanoparticles shaped as hexagonal plates exhibited higher AUC and longer MRT values and superior antitumor efficacy compared to spherical or rod-shaped nanoparticles [49]. These results suggest that a nanosilicate carrier with a sustained release profile of PTX may increase bioavailable PTX and tumor targeting and, thereby, have therapeutic potential. Our PSCT formulation is based on spherical mesoporous silica nanoparticles with a small pore diameter (2.54 nm), which retains several important advantages, including sustained PTX release (57.6% for 48 h) and increased tumor-specific PTX accumulation. Notably, spherical mesoporous silica nanoparticles can achieve efficient tumor penetration when properly sized (e.g., 100–200 nm) and offer well-established in vivo safety and formulation scalability [50]. The PSCT and SMB7 nanosilicate carriers in this study exhibited limited systemic toxicity during the treatment period. Therefore, our findings highlight that appropriately engineered spherical mesoporous silica nanoparticles with small pore diameters, such as PSCT in this study, can prolong drug release, enhance intracellular accumulation, exhibit potent anticancer effects, and reduce systemic toxicity.

Despite advancements in subcutaneous injection of PTX formulations, significant challenges remain in balancing injectability with therapeutic efficacy. While improving drug stability, high-viscosity formulations can cause necrosis at the injection site or inconsistent absorption due to various tissue characteristics. This phenomenon is exacerbated in individuals with a higher body mass index or compromised skin integrity. For instance, studies have shown that flow rates exceeding 10 mL/min at viscosities > 50 cP can induce tissue fracturing, thereby altering drug distribution and bioavailability [51]. Future investigations should emphasize the co-development of devices and formulations, such as thinner polymers or hyaluronidase analogs, to reduce viscosity without compromising payload capacity [51,52]. Additionally, long-term local toxicity profiles remain unexplored. While preclinical models have reported manageable profiles, the impact of chronic subcutaneous injection on tissue fibrosis or immune cell activation in humans remains unclear [53]. Rigorous pharmacokinetic–pharmacodynamic studies are necessary to correlate tumor exposure with therapeutic efficacy and tolerability [52,54].

## 5. Conclusions

PTX-loaded mesoporous silica nanoparticles combined with CUR and TPGS significantly improved anticancer efficacy in B16F10 melanoma-bearing mice. The average tumor growth size of the PSCT administrated group was reduced compared to the PTX + CUR administrated group by a factor of 5.5, from 1238 ± 177 mm^3^ to 224 ± 64.9 mm^3^. Compared to the control group, growth size was reduced by 14.9 times. PTX embedded SMB7 nanoparticle enhanced PTX solubility by 35.3-fold, as well as 8.75-fold enhanced drug release. The strong reciprocal correlation between PTX uptake and IC_50_ levels (r = 0.8939, *p* = 0.0408) indicated that the increased intracellular accumulation by overcoming P-gp-mediated efflux resulted in better therapeutic efficacy. This PSCT formulation induced robust apoptosis and pronounced microtubule disruption. PSCT formulation outperformed free PTX and PS formulations alone by 56% and 17% of radius decrease in the 3D-spheroid cytotoxicity test. The synergistic activity of CUR and TPGS effectively addressed increased tumor-specific accumulation while minimizing systemic toxicity. Thus, PSCT represents a promising nano-based therapeutic strategy for improving the treatment outcomes of drug-resistant melanoma. Further research should address long-term stability and optimize the clinical feasibility of subcutaneous delivery.

## Figures and Tables

**Table 1:** Pharmacokinetic parameters of PTX and CUR in B16F10 melanoma-bearing mice following subcutaneous administration of PSCT, PSC, PTX + CUR at a PTX dose of 2.5 mg/kg, CUR of 20 mg/kg, and TPGS 60 mg/kg.

Treatment	Parameters	PTX + CUR	PSC	PSCT
PTX 2.5 mg/kg	C_max_ (ng/mL)	53.98 ± 5.66	77.06 ± 7.58	62.96 ± 14.91
	AUC_last_ (h·ng/mL)	201.8 ± 92.92	301.2 ± 69.88	254.8 ± 18.15
	AUC∞ (h·ng/mL)	338.1 ± 43.41	444.6 ± 108.3	374.9 ± 25.64
	T_1/2_ (h)	3.44 ± 0.92	4.07 ± 1.38	4.40 ± 0.98
	T_max_ (h)	1.13 ± 0.55	0.82 ± 0.70	1.19 ± 0.82
	MRT (h)	2.28 ± 0.80	2.98 ± 0.73	3.10 ± 0.31
CUR 20 mg/kg	C_max_ (ng/mL)	102.7 ± 3.93	102.1 ± 55.12	246.0 ± 53.74 *
	AUC_last_ (h·ng/mL)	188.3 ± 31.97	198.2 ± 35.06	404.0 ± 75.65 *
	AUC∞ (h·ng/mL)	242.6 ± 10.54	210.8 ± 28.37	410.9 ± 75.55 *
	T_1/2_ (h)	1.43 ± 0.17	2.95 ± 1.82	1.35 ± 0.25
	T_max_ (h)	0.82 ± 0.70	0.60 ± 0.20	0.55 ± 0.25
	MRT (h)	2.28 ± 1.04	2.24 ± 0.83	1.49 ± 0.18

C_max_, maximum plasma concentration; AUC_last_ and AUC∞, area under the curve from time zero to last or infinity; T_1/2_, half-life; T_max_: time to reach C_max_; MRT, mean residence time. The data are represented as the mean ± SD (*n* = 5). * *p* < 0.05, compared with the PTX + CUR group.

## Data Availability

The original contributions presented in this study are included in the article. Further inquiries can be directed to the corresponding authors.

## Abbreviations

The following abbreviations are used in this manuscript:

Abbreviation	Full Term
AUC	Area Under the Curve
CUR	Curcumin
C_max_	Maximum Plasma Concentration
DAPI	4′,6-diamidino-2-phenylindole
DMEM	Dulbecco’s Modified Eagle Medium
DMSO	Dimethyl Sulfoxide
DSC	Differential Scanning Calorimetry
EPR	Enhanced Permeability and Retention effect
FBS	Fetal Bovine Serum
FT-IR	Fourier-Transform Infrared Spectroscopy
IC_50_	Half-Maximal Inhibitory Concentration
%ID/g	percentage of the injected dose per gram of tissue
IS	Internal Standard
JNK	c-Jun N-terminal Kinase
LC-MS/MS	Liquid Chromatography–Tandem Mass Spectrometry
MMP-9	Matrix Metalloproteinase-9
MRT	Mean Residence Time
MTT	3-(4,5-dimethylthiazol-2-yl)-2,5-diphenyltetrazolium bromide
NF-κB	Nuclear Factor-kappa B
PARP	Poly(ADP-ribose) Polymerase
PBS	Phosphate-Buffered Saline
P-gp	P-glycoprotein
PS	PTX-loaded SMB7 formulation
PSC	PS with Curcumin
PSCT	PS with Curcumin and TPGS
PST	PS with TPGS
PSV	PS with verapamil
PTX	Paclitaxel
ROS	Reactive Oxygen Species
SC	Subcutaneous
SEM	Scanning Electron Microscopy
SMB7	Smart Mesoporous Ball 7
TIMP-2	Tissue Inhibitor of Metalloproteinases-2
TPGS	D-α-Tocopherol Polyethylene Glycol 1000 Succinate
XRD	X-ray Diffraction
